# Facilitators to the continuous abuse of tramadol among the youth: A qualitative study in Northern Ghana

**DOI:** 10.1002/nop2.353

**Published:** 2019-07-30

**Authors:** Abdul‐Ganiyu Fuseini, Alhassan Afizu, Yakubu H. Yakubu, Gilbert Nachinab

**Affiliations:** ^1^ Department of Nursing, School of Allied Health Sciences University for Development Studies Tamale Ghana; ^2^ Nursing and Midwifery Training College Kpembe Ghana; ^3^ Intensive Care Unit Tamale Teaching Hospital Tamale Ghana; ^4^ Department of Midwifery, School of Allied Health Sciences University for Development Studies Tamale Ghana

**Keywords:** abuse, desirable, effects, physical, psychological, tramadol, undesirable, youth

## Abstract

**Study aim:**

Considering the alarming rate at which young people abuse tramadol, as evidenced by the numerous media reports on the subject, this qualitative study was conducted to explore the facilitators to the abuse of tramadol by young people.

**Design and methods:**

A qualitative exploratory descriptive design was employed in conducting the study. A total of 18 participants were purposively sampled. Data for the study were collected through two focused group discussions and three in‐depth‐interviews. Thematic analysis was used to analyse the data.

**Results:**

The findings of the study revealed four main themes. These themes were initiating factors of abuse; desirable physical effects; desirable psychological effects; and undesirable effects. It was revealed that many young people initially get into tramadol abuse because of peer pressure, curiosity or post‐traumatic addiction. However, they often continue the practice for various physical and psychological gratifications including euphoria, attentiveness, high energy levels, pain relief and improved sexual performance. The study also revealed some unpleasant side effects of tramadol abuse such as severe vomiting, loss of appetite, seizures, emotional aloofness and irritability. Many of the participants in this study also expressed willingness to quit tramadol abuse because of social discrimination and the enormous side effects that come with the abuse of the drug.

## 1 INTRODUCTION

Drug abuse is as an age‐old problem that has taken a toll on society and health systems. Many people, particularly young people, engage in drug abuse for varied reasons, including physical, psychosocial, educational, political and moral gains. The past decade has witnessed changing trends in the patterns of drug and substance abuse, with tramadol emerging as a leading drug that is widely patronized in the West African sub‐region (Oraegbune, Adole, & Adeyemo, 2017; Wakil & Ibrahim, 2017). Tramadol is an analgesic with an opioid‐like effect when taken orally (Global Commission on Drug Policy, 2017; WHO, 2017). It is primarily prescribed to treat mild to severe pain in both acute and chronic conditions (Abdel‐Hamid, Andersson, Waldinger, & Anis, 2016; Grond & Sablotzki, 2004). According to Olsson, Öjehagen, Brådvik, Kronstrand, and Hakansson (2017), approximately 32% of adolescents in Malmö, Sweden, abuse tramadol. While the level of abuse of tramadol is well documented elsewhere, estimates on the level of abuse of the drug are unclear in Africa due to the lack of studies on the epidemiological statistics of tramadol abuse in the continent. However, there is evidence of a growing trend of abuse of tramadol in most African countries, particularly Togo, Ghana, Libya and Egypt among others (WHO, 2017). Data available suggest that the annual seizures of tramadol in sub‐Saharan Africa have risen from 300 kg in 2013 to more than 3 tons in recent years, with Nigeria, Ghana, Togo, Sierra Leone, Cameroon and Côte d’Ivoire as the major transit or destination countries for tramadol (Salm‐Reifferscheidt, 2018). In 2015, nearly 70% of people treated in a state addiction facilities in Egypt were addicted to tramadol (Salm‐Reifferscheidt, 2018). Again, in neighbouring Nigeria, tramadol abuse has a prevalence rate of approximately 54.4%, with over 91% of these dependants obtaining the drug without prescriptions (Wakil & Ibrahim, 2017). Epidemiological data on the abuse of tramadol in Ghana are not readily available. However, reports from the Food and Drugs Authority (2018) posit that the proliferation and abuse of tramadol in higher doses continues to remain a major public health concern in the country (Food & Drugs Authority, 2018).

## 2 BACKGROUND

In Ghana and most countries in the West African sub‐region, tramadol is not on the list of controlled substances regulated by the Food and Drugs Authorities, because it is believed to have a low abuse potential compared with the prototypic opioids such as morphine (Salm‐Reifferscheidt, 2018; WHO, 2017). This has made the drug readily available in pharmacies, chemical shops and the black market and can be acquired without a prescription. Although the significance of tramadol abuse potential has been questioned (Adams, Dart, & Knisely, 2005; Salm‐Reifferscheidt, 2018; WHO, 2017), other evidence suggests that the drug has a clear risk of producing high abuse and dependence even among people without a history of substance abuse (Ferrari, Tiraferri, Palazzoli, & Licata, 2013; Stoehr, Essary, Ou, Ashby, & Sucher, 2009; Zhang & Liu, 2013). There have been several reports on the side effects of tramadol, especially when taken in higher doses (Oguntona & Adelowo, 2013; Zhang & Liu, 2013). These side effects, among others, include nausea and vomiting, constipation, sweating, dizziness, seizures and postural hypotension among others (Jovanović‐Cupić, Martinović, & Nesić, 2006; Oguntona & Adelowo, 2013; Zhang & Liu, 2013). In spite of the enormous side effects, many people continue to abuse tramadol for physical, psychosocial and sexual reasons. There has been evidence on the positive effects of tramadol on the sexual performance of men (Abdel‐Hamid et al., 2016; Wong & Malde, 2013). According to Salm‐Reifferscheidt (2018), tramadol mixed with energy drink is a common remedy for enhanced sexual performance among men in neighbouring Benin and Nigeria. Wong and Malde (2013) also assert that tramadol on‐demand results in a significant improvement in mean intravaginal ejaculatory latency time and an improvement in partner sexual satisfaction scores. While efforts are made by researchers to further illumine the rationale for the abuse of tramadol elsewhere, little is known about the facilitators to the abuse of tramadol among young people in Ghana, as a result of insufficient data on the dynamics of tramadol abuse in the country. However, the researchers as citizens and residents of Northern Ghana have observed with worry, the alarming rate at which young people of the country abuse the drug, as evidenced by various media reports. Again, anecdotal evidence in the Tamale Metropolis, where the study was conducted, suggests an alarming rate of tramadol abuse among young people of the town, for physical and psychosocial gains. However, this evidence has not been explored empirically to afford any scientific statement on the matter. It is for this reason that the researchers conducted this qualitative exploratory study to explore the facilitators to the abuse of tramadol among young people in the Tamale Metropolis of the Northern Region of Ghana. To the best of our knowledge, this is the first qualitative study that explored the reasons for the abuse of tramadol among young people in Ghana.

## 3 METHODS AND MATERIALS

The researchers employed the qualitative exploratory descriptive design in the present study. Qualitative studies involve the study of individuals or groups in their natural settings, attempting to make sense of phenomena in terms of the meanings the individuals bring to them (Howitt, 2010; Lambert & Lambert, 2012). The goal of a qualitative exploratory descriptive study is a comprehensive summarization in everyday terms, of specific events experienced by individuals or groups of individuals (Lambert & Lambert, 2012). To achieve a broad understanding of the facilitators to the continuous abuse of tramadol among young people in the Tamale Metropolis, a qualitative descriptive design was the best approach for the study.

**Figure 1:**
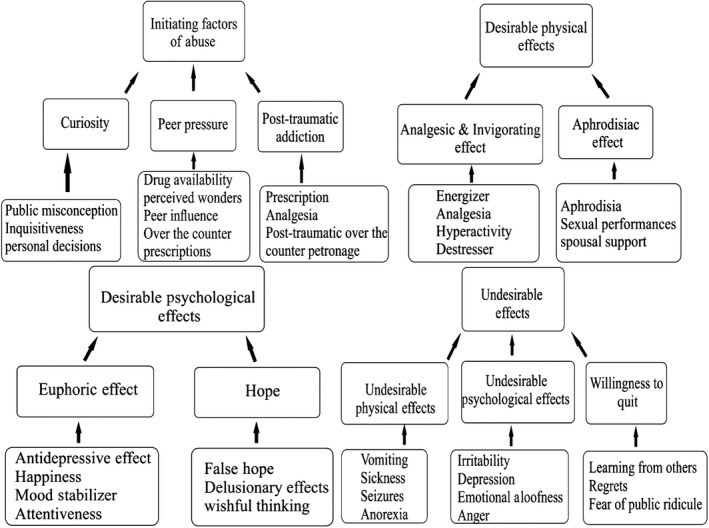
A thematic map indicating the development of themes and subthemes

### 3.1 Study setting

The study was conducted in Tamale, which is officially called Tamale Metropolitan Area and is the capital town of the Northern Region of Ghana. Tamale is Ghana's fourth largest city with a population of 360,579 people, according to the world population review (World Population Review, 2018) and is the fastest‐growing city in West Africa (Ghanaweb, 2014). Males constitute 49.7% and females represent 50.3%. In addition, the population of the metropolis is youthful with nearly 36.4% of the population below the age of 15 years. Of the population 11 years and above, 60.1% are literates and 39.9% are non‐literates (Ghana Statistical Service, 2012). The town is located 600 km north of Accra, the capital of Ghana, precisely in the Kingdom of Dagbon. More than 80% of the inhabitants are Dagombas. Other large groups living here include the Dagarbas, Mamprusis and Akans. Due to its central location, Tamale serves as a hub for all administrative and commercial activities in the Northern region. It also is the political, economic and financial capital of the Northern region.

**Table 1:** Demographic characteristics of participants

Variables	*N*	%
Gender		
Male	15	83.3%
Female	3	16.7%
Age		
18–25 years	13	72.2%
26–35 years	5	27.8%
Level of education		
No formal education	2	11.1%
Basic education	11	61.1%
Secondary education	5	27.8
Tertiary education	N/A	
Marital status		
Married	3	16.7%
Not married	15	83.3
Religion		
Muslim	16	88.9%
Christian	2	11.1%
Duration of abuse of tramadol		
<2 years	1	5.6%
≥2 years	15	83.3%
Unknown	2	11.1%

Abbreviation: N/A: Not applicable.

### 3.2 Sample and sampling procedure

Purposive sampling technique was employed in recruiting the sample for the present study. A total of 18 participants who are dependent on tramadol were recruited for the study. The sample size was determined by data saturation. Saturation in qualitative research is achieved when new emerging themes are not forthcoming (Houghton, Casey, Shaw, & Murphy, 2013a). In recruiting the sample, the researchers sought approval from the management of the Tamale Teaching Hospital to use the psychiatric unit of the hospital as an outlet for the recruitment of the sample. The psychiatric unit of the Tamale Teaching Hospital is the only unit in the Tamale Metropolis that provide medical and rehabilitation services to people with drug addiction. Following approval by the authorities of the hospital, the researchers visited the psychiatric unit of the hospital and enlisted the support of colleague nurses in the unit for the recruitment of the sample. The colleague nurses contacted prospective participants on behalf of the researchers to inform them about the study and to create a link between the participants and the researchers. Following this, the researchers contacted the participants who met the inclusion criteria of being 18 years or older, documented medical diagnosis of tramadol dependence, ability to communicate in English language or Dagbani (a local dialect) and willingness to participate, for rapport building. Participants who met the inclusion criteria and agreed to take part in the study were subsequently recruited after signing of an informed consent. To achieve maximum variation with the sample of the study, the researchers recruited both male and female, but information‐rich participants whose dependence on tramadol was a public knowledge in the research setting. These participants gave vivid accounts of their reasons for the continuous abuse of tramadol.

### 3.3 Data collection methods and procedure

Data for the study were gathered through focus group discussions and face‐to‐face in‐depth interviews. The researchers conducted two focus group discussions involving eight and seven male participants in the first and second focus groups discussions, respectively. Each focus group was conducted by two researchers (an interviewer and a note‐taker). The focus groups lasted for approximately 54 min and 1.5 hr for the first and second focus group discussions, respectively. The participants for the focus group discussions were mainly males. This was to create an enabling environment where participants could express themselves freely without fear. Each focus group was recorded on an audio tape recorder with permission from the participants. To ensure confidentiality and anonymity, numbers were assigned to each participant prior to the focus group discussions and demographics data were collected before the discussions. In addition to the focus groups discussions, the first author conducted three face‐to‐face in‐depth interviews involving female participants to get the perspectives of the female gender on the objective of the study. The interviews lasted for approximately 33–42 min and were equally recorded on an audio‐recorder with participants’ permission. The questions for the interviews and focus groups were developed based on the objectives of the study. Some of the questions that were asked during the data collection were as follows: could you please tell me what you know about tramadol? What motivated you to start taking tramadol? Could you please share with me how you feel, physically after taking tramadol? How would you describe the impact/effect of tramadol on your work or daily activities? How do you feel deep inside you any time you take tramadol? How do you think you relate with people when you are under the influence of tramadol? The authors used semi‐structured interview guides for the data collection to allow participants to express their thoughts. Field notes of all non‐verbal communications were taken during the data collection process to ensure that every aspect of the data was captured and also to help in the analysis.

### 3.4 Data analysis

Data from the study were analysed concurrently with data collection using thematic analysis. At the end of each focus group discussion or interview, the authors manually transcribed verbatim the audio tape recording of the discussion or interview. The transcribed data were checked for accuracy by reading over it and at the same time, listening to the audio tape recordings by a different researcher. After the transcription, the data were analysed using the six stages of thematic analysis proposed by Braun and Clarke (2006). These stages are as follows: Data Familiarization; Initial Coding Generation; Search for Themes Based on Initial Coding; Review of Themes; Theme Definition and Labelling and Report Writing, respectively. The researchers familiarized themselves with the content of the data by reading each transcript many times to gain a sense of the whole data. In addition, because of the close involvement of the researchers with the data collection and transcriptions, the data were very familiar to them even before the analysis. The second stage of the thematic analysis according to Braun and Clarke (2006) is the initial code generation. In generating the codes from the data, the texts in the transcripts that were relevant to the objectives of the study were identified. These relevant texts were highlighted in Microsoft Word on the computer for easy identification during coding. While the transcripts were read, the authors searched for similar ideas, thoughts and words within the data and these made up the codes. Identified codes were written against the lines of the transcripts where the codes were found on the right margin of the transcripts that was contained in a Microsoft Word document. The initial codes were revised in the light of things that appeared later in the transcripts. Each transcript was handled in this same manner and new codes that emerged during the coding process were added until all the entire transcripts were coded.

The third stage of the data analysis (Searching for Themes Based on Initial Coding) involved more interpretation and inductive reasoning. The relationships between the codes were analysed by the authors and the codes were grouped based on their relationships. Following this, constructs which embraced the various groups of the initial codes were identified and these formed the subthemes. In the fourth stage of the thematic analysis according to Braun and Clarke (2006), (Review of Themes), the researchers reviewed all the subthemes to establish possible relationships between the various subthemes. Subsequently, subthemes that shared similar characteristics were further grouped into main themes based on the relationship that existed between them. The main themes were then labelled based on the general consensus of the researchers, by choosing concepts that embraced all the subthemes in each main theme (Theme Definition and Labelling). Again, the last stage of the thematic analysis process according to Braun and Clarke (2006) is report writing. This is where the authors presented the results of the study, supported with verbatim codes. Throughout the analysis, the authors moved backwards and forward between the data, the codes, as well as between the developing subthemes until the main themes were well defined.

### 3.5 Trustworthiness

The researchers employed several measures to ensure the validity of the findings of the study. To achieve Transferability (Cope, 2014) in the study, the researchers gave a vivid description of the research setting and also employed a sample size that was large enough to yield data saturation. To achieve credibility of the data, the researchers purposefully recruited participants that met the inclusion criteria and could provide in‐depth information on the reasons for their continuous abuse of tramadol. Again, the authors spent sufficient time on the field to gain a fuller and deeper understanding of the participants’ experiences with the drug. Credibility was further enhanced in the study through member‐checking (Houghton et al., 2013a); Transcripts of the interview were taken back to the participants and explained to them in the local dialect for comments and verifications before conclusions were drawn from the data. In achieving dependability in the study, the researchers maintained an audit trail by giving a transparent and in‐depth description of the research design, background of participants and the methods used in collecting and analysing the data. The authors also employed the services of other researchers who were not involved in the data collection, to examine and make comments on the processes and findings of the study. The purpose was to evaluate the accuracy and assess whether the findings, interpretations and conclusions were supported by the data. To address any conflict of interest, the researchers reflected on their own biases and prejudices and bracketed and controlled them before the data were collected.

### 3.6 Ethical considerations

The study was reviewed and approved by the Research Ethics Committee of the Tamale Teaching Hospital (TTH/R&D/SR/137). In addition, the management of the hospital (TTH) gave approval to use the psychiatric unit of the hospital as an outlet for the recruitment of the sample. The purpose, objectives and any potential benefits and risks for participating in the study were also explained to participants in the local dialect (Dagbani) or English a few days prior to data collection. This allowed participants enough time to consider their participation. Informed consent was obtained from each participant that met the inclusion criteria and agreed to take part in the study. The participants were also informed that they could decline to participate or withdraw from the study even after they had signed the consent form without any consequences. The permission to record the interviews was sought from each participant who agreed to participate in the study. Again, identifiable demographic details of the participants such as names of the participants were replaced with numbers to ensure anonymity and confidentiality. Data from the study were kept under lock and in the first author's office and only the researchers had access to the data.

## 4 RESULTS

Most participants were males (83.3%) while others were females (16.7%). This was influenced by the approaches used in the recruitment of the sample and data collection. Many of the participants were also between the ages of 18 and 25 years (72.2%) and just a few participants were between the age brackets of 26 and 35 years (27.8). On level of education, most participants 11 (61.1%) had basic education, 5 (27.8%) had secondary education and 2 (11.1%) had no formal education. None of the participants had tertiary institution. Only 3 (16.7%) of the participants were married and the rest were single. Again, most participants 16 (88.9%) were Muslims while just 2 (11.1%) were Christians. This may be attributed to the fact that the research setting (Tamale Metropolis) is a Moslem‐dominated geographical area. On the duration of abuse, only 1 (5.6%) of the participants reported abuse of tramadol for less than 2 years, while most participants 15(83.3%) reported dependence on the drug for 2 years of more. Two of the participants (11.1%) could not recall the duration for their abuse of tramadol (Table 1).

The findings of the study revealed four main themes. These themes were initiating factors of abuse; desirable physical effects; desirable psychological effects and undesirable effects (Figure 1).

### 4.1 Initiating factors of abuse

The first objective of the study was to explore the factors that ushered the participants into the abuse of tramadol. The participants gave vivid accounts of how they were introduced to tramadol. Most participants identified peer pressure as the root cause of their addiction to tramadol while others cited curiosity and post‐traumatic addiction as the factors they got them introduced to the drug. The subthemes that were identified under this main theme are discussed as follows:

#### 4.1.1 Peer pressure

Most participants said they were introduced to tramadol by their friends. Some of the participants reported that the drug was recommended to them by their friends as an effective cure for bodily pains while others indicated that tramadol was recommended to them by friends as a mood‐stabilizing agent that could help light up their mood. This is supported in the following quotations from the participants:

I started taking tramadol when I woke up one day and had some neck pains and waist pains. So I complained to a friend who recommended it for me. And when I took it, it really worked for me. (21‐year‐old male)

Another participant said:

A friend told me that the drug was very good. That if I take it I would be feeling very “normal.” So I should try it and see. At first I resisted, but later he convinced me. So for me it was a friend who convinced me to take it the first time. (19‐year‐old male)

A few of the participants also reported that they were encouraged to continue with the intake of the drug after experiencing enormous side effects with their first intake, with the hope of achieving the desirable effects in future. This is what one of the participants said:

When I took it for the first time, I felt very weak and dizzy. But my friends told me that probably my system was not yet used to the drug, so I should continue taking it until my system adjusts to it. (18‐year‐old male)

#### 4.1.2 Curiosity

It was also observed from the data gathered that some of the participants started the abuse out of curiosity. There have been a lot of campaigns against the abuse of tramadol on both electronic and print media at the research setting. These campaigns further propagate the name of the drug as there are a lot of public misconceptions about the possible benefits of the drug. As a result of the above, some of the participants of the study verbalized that they first took tramadol because they wanted to confirm all the news they heard about the drug. One participant said:

People talk a lot about that drug you know. So, I just wanted to explore the wonders of it cos I overheard my guy and his friends praise that drug a lot. So, I think that’s how it all started. Yes! I just feel great each time I take it. (21‐year‐old female)

One of the participants also indicated that he just wanted to try the drug and see having heard a lot about what it could do from friends. He said:

……you know, when you hear about something, especially from your friends, you want to kill your curiosity by trying for yourself. So, I would say…..that was how it all began. (24‐year‐old male)

#### 4.1.3 Post‐traumatic addiction

In addition to curiosity and peer pressure, two of the participants also mentioned that they got addicted to tramadol after the drug was prescribed to cater for their pain at the hospital following a road traffic accident. A participant said:

I started using tramadol when I injured my leg in an accident. I was in so much pain at the hospital but when the nurse just gave me 2 tablets of tramadol, the pain was gone in no time and I fell asleep. By the time I woke up, they had dressed the wound. And when I left the hospital, I continued taking it and it has always been normal for me. (32‐year‐old male)

### 4.2 Desirable psychological effects

Another main theme that was identified from our data and that serves as one of the reasons for the continuous abuse of tramadol by the sample of our study was the desirable psychological benefits. The participants enumerated several benefits of the abuse of tramadol on their psychological health and well‐being. According to them, tramadol gives them hope, increases their mental attentiveness and makes them extremely happy. The subthemes that were identified under this category are discussed as follows:

#### 4.2.1 Euphoric effects

Most participants indicated that they become extremely happy each time they are under the influence of tramadol. As a result of its euphoric effects, a few of the participants also reported that they usually take the drug each time they have a bad day or when they are in a sad mood. This is supported in the following quotations from a section of the participants:

When you take it, you will just be full of happiness. You wouldn’t have any problem with anybody. (22‐year‐old male)

Sometimes…. Just like my colleagues have indicated, you may get sad because of something and you just try to make yourself happy, so you go in for it because it helps. (25‐year‐old male)

In addition to the euphoric effect of the drug as reported by most participants, a few of the participants also verbalized that the drug increases their attention levels and makes them highly focused on whatever they do without recourse to environmental detractors This was what one of the participants said:

When I take it, I can be online chatting with people from morning till evening and I will not even hear the phone bell, or even maybe the light or something. So, I take it when I have serious stuffs to do, you get what I mean? (20‐year‐old male)

#### 4.2.2 Hope

Another subtheme that was identified under this category was hope. In addition to the euphoric effect, some of the participants also added that they become hopeful of a better future and feel as though they have won a lottery, each time they are under the influence of the drug:

For me, when I first took it, it didn’t cause any negative reaction in me. It rather gave me excitements because, I felt as though I was expecting a life changing good news or had won a jackpot and expecting voyage of vehicles from a trade expedition. (19‐year‐old male)

### 4.3 Desirable physical effect

This major theme describes the participants’ physical experiences with tramadol abuse. The participants cited several desirable physical effects as the reasons for the sustained abuse of tramadol. The subthemes that arose from the data under this category were analgesic and invigorating effect and aphrodisiac effect.

#### 4.3.1 Analgesic and invigorating effect

Most participants reported that they use tramadol to de‐stress themselves when they are fatigued or stressed up after the day's work. A few others indicated that they used the drug each time they suffered any pain. Again, some of the participants also verbalized that tramadol enhances their performance at work by bolstering their energy and enabling them to be able to perform their duties without stress. In spite of the numerous campaigns against the abuse of tramadol, a few of the participants still see tramadol abuse as important to their lives. The above report is supported by the following quotations from a selection of the participants:

Tramadol is a pain killer you know. Some of us, we do hard work. So, after the work, when you take the drug, it takes away the fatigue and you feel normal. (35‐year‐old male)

For me, I am a farmer, so when I take it and go to farm, I can work for several hours, like from 9:00 a.m. to 6:00 p.m. So, for the drug, it is some few people who have caused some disaffection for it, otherwise it is not a bad thing. (26‐year‐old male)

#### 4.3.2 Aphrodisiac effect

Another desirable physical effect of tramadol which serves as one of the reasons for the continuous abuse, as reported by participants of our study, is its sexual enhancing effect. Several participants of the present study mentioned that tramadol enhances their sexual performance and hence, a reason for the continuous abuse. Some of the participants also reported that their spouses endorse their use of the drug as it enables them to meet the sexual needs of these spouses. Two of the participants also indicated that their girlfriends often buy the drug for them, so it can improve their sexual performance. A participant shared his experience:

…..and also, for sexual performance; it is very powerful in that regard. If you take it, you can go for up to 40 minutes without coming. (22‐year‐old male)

He added:

Yeah! That’s just first round. As for the second round, you will just have to advise yourself and stop. And your erection will just remain solid, without relaxing. (22‐year‐old male)

Me too I have a girlfriend and…… she herself…. you know! I already mentioned that some girls actually buy it for their men, so for my girlfriend, sometimes I will sleep and wake up and find the drug on my table and I mean she is the one who brought it. So, I will just take it so I can become strong enough to give it to her as she wants. (29‐year‐old male)

### 4.4 Undesirable effects

Besides the desirable effects for which participants of the study abuse tramadol, the participants also shared their experiences on some of the adverse effects they endure as a result of the abuse. The subthemes that were identified under this main theme were undesirable physical effects, undesirable psychological effects and willingness to quit.

#### 4.4.1 Undesirable physical effects

The participants mentioned myriad of adverse physical effects they suffer each time they abuse tramadol. These adverse physical effects were anorexia secondary to profuse vomiting and seizures. The participants verbalized that they often lose appetite for food each time they take the drug, as a result of the vomiting that comes with the intake of the drug. The participants further indicated that besides soft drinks and water, anything they take by mouth would be vomited out so long as they are under the influence of tramadol. In addition to the above, a few of the participants also reported that the side effects were more pronounced in their first abuse of the drug but subsided in subsequent abuses. Some participants said:

When I first took it, it made I vomited a lot. And I was feeling sick, as if I was having malaria and nothing stayed in my stomach that day. I vomited everything out. Now it’s better but…….. (22‐year‐old female)

One of the participants echoed:

For me, the moment I take it I can’t eat anything. Anything I put in my stomach, I will vomit it, because of that I don’t eat, because I know if I attempt, I will vomit everything. But usually I am able to take drinks or water. (19‐year‐old male)

Another adverse physical effect of tramadol reported by the participants was seizures. Although none of the participants involved in the study reportedly experienced a seizure as a side effect of the drug, most of them indicated that they had witnessed on several occasions, colleagues who suffered seizures as a result of the abuse of tramadol. Some of the participants identified higher doses of the drug as a possible cause of the seizure, while others were of the view that, the seizure comes about when the drug is taken on an empty stomach. Two said:

What he is saying is actually true because, for me it has never made me collapse, but I have watched someone else had a seizure and fell and started foaming at the mouth. So, it can be very hard. Even if you want to take, you should just take something small. (28‐year‐old male)

Another participant said:

In my observation, I want to advise those of us taking it, it is important to eat before taking it, you should not take it on an empty stomach, if not you would have problems. It can make you collapse, develop seizures, etc. But if you eat before taking it, that would not happen to you. (23‐year‐old male)

#### 4.4.2 Undesirable psychological effects

In addition to the undesirable physical effects of anorexia, nausea, vomiting and seizures, some of the participants in this study also reported several perceived emotional problems as part of the adverse effects of the abuse of tramadol. These emotional problems according to the participants include irritability, anger and emotional aloofness. The participants indicated that the drug makes them temperamental with the least provocation and also makes them lose interest in interacting with others in the society. These assertions are supported by the following:

Sometimes when I take it, I don’t like too much talking because I easily get irritated when someone worries me with unnecessary talk. (20‐year‐old male)

Another participant shared:

When I take it, I would be sitting down quietly because I don’t feel like talking to anyone. And when someone just plays with me a little, I lose my temper. I don’t want to mingle with anyone. (25‐year‐old male)

#### 4.4.3 Willingness to quit

Most participants of our study also expressed a desire to quit the abuse of the drug as a result of lessons learned from other colleagues who experienced the worse form of adverse effects of the drug (seizures). The participants were of the view that they needed to stop the abuse to avoid the possibility of a seizure as a side effect for the abuse of tramadol. One of the participants said:

Just like the seizures they have been talking about, I have seen a lot of people go through that and it can be very frightening. So, if you see it like that and you know that it is because he used tramadol, obviously you will want to stay away from it. (31‐year‐old male)

## 5 DISCUSSION

The main purpose of the study was to explore the reasons for the abuse of tramadol among young people in the Tamale Metropolis of Ghana. The first objective was to identify the circumstances under which the participants were introduced to the drug (tramadol). According to the data gathered, the participants got introduced to the drug by several factors. These factors were peer group pressure, curiosity and post‐traumatic addiction. Most participants reported that they were convinced by their friends to take tramadol for the first time while some few other participants reported that their first experience with tramadol was to satisfy their curiosity about the wonders the drug could perform, as widely believed by the public. In tandem with these findings, peer pressure and curiosity have been cited in literature as some of the main causes of tramadol abuse (El Wasify et al., 2018). A few (2) of the participants of our study also reported that, they got addicted to the drug after it was prescribed for them for the management of their pain at the hospital. This finding adds to the existing debate on the abuse potential of tramadol: In consonance with the finding of our study, several studies have reported on the high abuse potential of tramadol (Rougemont‐Bücking, Gamma, & Panksepp, 2017; Rougemont‐Bücking et al., 2017; Stoehr et al., 2009; Stoehr et al., 2009; Zhang & Liu, 2013) while other studies have identified tramadol as a drug with a low potential for abuse and dependency (Cicero et al., 1999; Knisely, Campbell, Dawson, & Schnoll, 2002; Radbruch et al., 2013). Future experimental studies involving large transcultural samples would help shed more light on the abuse potential of tramadol among the human population.

In addition, participants of the present study also enumerated several psychological gratifications they derive from the abuse of tramadol. According to the participants, these gratifications serve as the main reasons for their continuous use of the drug. The participants cited among others, hope, euphoria and attentiveness, as the main psychological gratifications they drive from the abuse of tramadol. Nearly all the participants indicated that tramadol abuse elevates their mood and makes them feel extremely excited. As a result of this antidepressant effect, some of the participants of our study often abuse the drug each time they get worried, as the drug gives them hope and makes them feel happy as if they have won a lottery, according to the participants. This finding supports existing literature on the antidepressant effect of tramadol (Kalra, Tayal, & Chawla, 2008; Ostadhadi et al., 2017; Rougemont‐Bücking et al., 2017; Tetsunaga et al., 2016). In a systematic review, Rougemont‐Bücking et al. (2017) reported the case of two people who witnessed a significant improvement in their mood after taking a prescription of tramadol for the management of their pain. In addition to the reported euphoric effect of tramadol, a few other participants of the current study also noted that the drug increases their attention levels and makes them highly focused and concentrated on whatever they do without recourse to environmental distractors. In line with this finding, Holgado et al. (2018), in a randomized control trial assert that, tramadol has an impact on stimulus processing related to sustained attention. Future studies should focus on shedding light on the antidepressant and increased mental concentration effects of tramadol among human beings.

Besides the psychological gratifications as discussed above, participants of the present study also cited several desirable physical effects as part of the reasons for their sustained abuse of tramadol. These desirable physical effects were invigorating effect, analgesic effect and aphrodisiac effect. Most participants reported that tramadol energizes them and as a result, they often take the drug either to de‐stress themselves after the hard day's work, or to get the energy to carry on with their daily activities without getting fatigued. In tandem with this finding, Holgado et al. (2018), in a randomized controlled trial demonstrated that tramadol enhances task performance. Again, some of the participants of the present study verbalized that they use tramadol for its potent analgesic effect. The participants indicated that they used the drug to manage their pain each time they suffered any pain. This finding corroborates the existing wealth of knowledge in literature, on the analgesic effect of tramadol (Attal, 2018; Sheikholeslami, Jamali, & Rouimi, 2016). More so, another desirable physical effect of tramadol that serves as one of the reasons for its continuous abuse by participants of our study is its aphrodisiac effect. Several participants mentioned that tramadol enhances their sexual performance and grants them the ability to satisfy the spouses. For this reason, the participants receive spousal support and endorsement for their abuse of the drug. Some of the participants reported that their spouses often support them financially in the purchase of the drug because of its sexual enhancing effect. In line with this finding, tramadol has been cited in past studies as a potent drug for its off label effect of increasing intravaginal ejaculatory latency time and for the treatment of premature ejaculation (Abdel‐Hamid et al., 2016; Kirby, Carson, & Coward, 2015). While the abuse of tramadol for its off label effect of enhancing sexual performance is widely reported in literature (Abdel‐Hamid et al., 2016; Kirby et al., 2015; Wong & Malde, 2013), a recent study by Farag et al. (2018) has also reported low sperm density, motility and vitality among male addicts of tramadol. Future randomized controlled trials would help throw more light on the efficacy and safety of the use of tramadol for it's off label aphrodisiac effect.

Besides the desirable effects of tramadol, the participants of our study also cited several adverse effects they endure as a result of the abuse of the drug. These adverse effects were irritability, anorexia secondary to profuse nausea and vomiting and seizures. Some of the participants reported that the drug makes them get temperamental with the least provocation and also makes them lose interest in interacting with others in society. There is lack of information on the effects of tramadol on the psyche and behaviour of abusers. Future studies should focus on the psychoactive effects of tramadol among tramadol‐dependent individuals. Again, participants of our study reported loss of appetite, induced by excessive vomiting and seizures as some of the adverse effects for the abuse of tramadol. Some of the participants indicated that besides soft drinks and water, nothing they ingest, stays in their stomach each time they are under the influence of tramadol. These findings are in agreement with the findings of previous studies on the common side effects of tramadol (Oguntona & Adelowo, 2013; Rahimi, Soltaninejad, & Shadnia, 2014). Although none of the participants in our study reported experiencing seizure as an adverse effect of the drug, most participants, however, reported that they had witnessed their colleagues suffered a seizure activity after an abuse of tramadol. The participants expressed desire to quit the abuse of the drug as a result of the adverse effects they experience and more importantly, as a result of lessons learnt from colleagues who have suffered seizures as a result of abuse of the drug. Future research should focus on evaluating the effectiveness of “learning from others” as an intervention for the control of tramadol abuse.

## 6 CONCLUSION

This study has brought to the fore the reasons for the widespread abuse of tramadol among the Ghanaian youth. Consistent with findings from other countries, this study identified curiosity, peer pressure and post‐traumatic addiction, as the main factors that often usher young people into the practice of tramadol abuse. Once they are introduced to the drug, young people remain hooked to it because of various physical and psychological gratifications that they gain from the drug. These gratifications, as revealed by the findings in this study include increased concentration and attentiveness, feelings of euphoria, relief from severe pain, increased energy levels and improved sexual performance. However, the study also revealed that tramadol abuse is actually not an all‐rosy affair, as it is usually associated with some equally worrying undesirable effects including severe vomiting, loss of appetite, seizures, irritability and social isolation.

One good revelation from this study is that many of the participants expressed willingness and desire to quit the practice, having taken cognizance of the damage that tramadol abuse could cause them. Therefore, government and the private sector should step up efforts on the campaign against tramadol abuse. More importantly, government should set up rehabilitation centres in all ten regions of Ghana to provide professional guidance and rehabilitation to those who are willing to quit.

## CONFLICT OF INTEREST

The authors have no conflict of interest.
